# Ras-driven cancer cells can scavenge exogenous lipids to support their proliferation

**DOI:** 10.1186/1753-6561-6-S3-P25

**Published:** 2012-06-01

**Authors:** Jurre J Kamphorst, Justin R Cross, Jing Fan, Wenyun Lu, Eileen White, Craig B Thompson, Joshua D Rabinowitz

**Affiliations:** 1 Lewis-Sigler Institute for Integrative Genomics and Department of Chemistry, Princeton University, Princeton, New Jersey 08544, USA; 2 Cancer Biology and Genetics Program, Memorial Sloan-Kettering Cancer Center, New York, NY 10065, USA; 3 The Cancer Institute of New Jersey, New Brunswick, New Jersey 08903, USA; 4 University of Medicine and Dentistry of New Jersey, Robert Wood Johnson Medical School, Piscataway, New Jersey 08854, USA; 5 Department of Molecular Biology and Biochemistry, Rutgers University, Piscataway, New Jersey 08854, USA

## Background

Lipids are an important structural component of the cell, making up the cell’s membranes. Cancer cells need lipids in large quantities to enable their rapid proliferation. Here we aim to quantitatively evaluate the routes by which cancer cells acquire lipids (*de novo* synthesis, uptake from environment) under various conditions (specific oncogenic activations, nutrient availability). We developed a mass spectrometry-based method to quantitate the relative contributions of the various fatty acid acquisition routes and applied this and other methods to study fatty acid metabolism in cell lines, both *in vitro* and *in vivo.*

## Materials and methods

Analysis of fatty acid acquisition routes was done by combining ^13^C-tracers with liquid-chromatography mass spectrometry (LC-MS): cells were grown in medium with uniformly labeled ^13^C-glucose and ^13^C-glutamine. Fatty acid samples were generated by saponifying (hydrolyzing) whole cell lipid extracts from these cultured cells, and were analyzed by high resolution LC-MS. Fluxes and relative contributions of fatty acid metabolic events (de novo synthesis, uptake, elongation, desaturation) were computed from the observed isotopic patterns. In addition, intact lipid (lipidomics) measurements were done by high resolution LC-MS. These approaches were applied to multiple cell lines with a variety of oncogenic lesions: isogenic transformed baby mouse kidney cell lines (BMK) with either Akt or H-Ras pathway activation, A549 (K-Ras mutant), MDA-MB-468 (PTEN null), and other cancer cell lines. The main findings were corroborated in xenograft experiments.

## Results

Determination of fatty acid acquisition routes in an isogenic model with either Akt or Ras pathway activation demonstrated that Akt induces de novo fatty acid synthesis, whereas Ras decreases it. This was most evident for the mono-unsaturated fatty acid oleate (C18:1), of which 96% was produced de novo in Akt-driven cells but only 57% in Ras-driven cells. We confirmed that Akt pathway activation leads to elevated levels of stearoyl-CoA desaturase 1 (SCD1), a key enzyme in the synthesis of oleate, and that Ras pathway activation leads to increased uptake of exogenous lipids. We hypothesized that SCD1 inhibition would therefore be particularly toxic to the Akt-driven cells. We confirmed that Akt activated cells were significantly more sensitive to SCD1 inhibition than those with Ras activation. Growth of Ras-driven cells was unaffected by SCD1 inhibition for multiple doublings, after which the cells stopped growing. We found that growth inhibition coincided with depletion of lysolipids in the media. This Ras-dependent phenotype was also observed in A549 cells (K-Ras mutant) and other cell lines. A xenograft experiment with A549 cells demonstrated that tumor growth in mice on a diet containing abundant oleate was not affected by SCD1 inhibition, whereas tumor growth was significantly inhibited in mice fed with an oleate poor diet.

## Conclusions

This work contributes to explaining at a molecular level how diet may impact tumor progression. In addition, the improved understanding of how cancer cells acquire their lipids will aid future efforts that target lipid metabolism as an anti-cancer strategy.

